# Teleonomy: Revisiting a Proposed Conceptual Replacement for Teleology

**DOI:** 10.1007/s13752-022-00424-y

**Published:** 2023-01-20

**Authors:** Max Dresow, Alan C. Love

**Affiliations:** 1 Minnesota Center for Philosophy of Science, University of Minnesota, Minneapolis, MN USA; 2 Department of Philosophy & Minnesota Center for Philosophy of Science, University of Minnesota, Minneapolis, MN USA

**Keywords:** Adaptation, Evolution, Goal-directedness, Mechanism, Purpose

## Abstract

The concept of teleonomy has been attracting renewed attention recently. This is based on the idea that teleonomy provides a useful conceptual replacement for teleology, and even that it constitutes an indispensable resource for thinking biologically about purposes. However, both these claims are open to question. We review the history of teleological thinking from Greek antiquity to the modern period to illuminate the tensions and ambiguities that emerged when forms of teleological reasoning interacted with major developments in biological thought. This sets the stage for an examination of Pittendrigh’s (Adaptation, natural selection, and behavior. In: Roe A, Simpson GG (eds) Behavior and evolution. Yale University Press, New Haven, pp 390–416, 1958) introduction of “teleonomy” and its early uptake in the work of prominent biologists. We then explore why teleonomy subsequently foundered and consider whether the term may yet have significance for discussions of goal-directedness in evolutionary biology and philosophy of science. This involves clarifying the relationship between teleonomy and teleological explanation, as well as asking how the concept of teleonomy impinges on research at the frontiers of evolutionary theory.

## Introduction

In a 1987 contribution to *Perspectives in Ethology*, behavioral scientist Nicholas Thompson observed that, “the use of the word ‘teleonomy’ to refer to the study of goal-directed processes…has not caught on” (Thompson 1987, p. 260). Several decades later this remains the case. One hears relatively little about “teleonomy” these days, although a recent conference sponsored by the Linnean Society speaks to renewed interest in the concept.^1^ Still, “teleonomy” had a moment—not in the 1980s, when Thompson wrote, but in the 1960s and 70s, when prominent biologists like Ernst Mayr, George C. Williams, and Jacques Monod all advocated its use. The question this article attempts to answer is *why*? Why did teleonomy catch on when it did, and why was it subsequently marginalized on its home turf of evolutionary biology? Moreover, what are the prospects for its revival, especially in the context of a revised and expanded evolutionary theory (e.g., Laland et al. 2015)?

To answer these questions, we start by reconstructing the background to Colin Pittendrigh’s coinage of the term “teleonomy” in 1958. This takes us all the way back to ancient Greece, where teleological thinking received its earliest and most influential articulations. Then, we trace the career of different strands of teleological thinking to illuminate the tensions and ambiguities that emerged when these interacted with major developments in biological thought. Coming to the 20th century, we examine Pittendrigh’s chapter in *Behavior and Evolution* (1958), which introduced “teleonomy” into the biological lexicon. We also discuss its early uptake in the work of Mayr, Williams, and Monod, and suggest several reasons for its marginalization. Finally, we analyze the significance of “teleonomy” for contemporary discussions of goal-directedness in evolutionary biology and philosophy of science. This involves clarifying the relationship between teleonomy and teleological explanation, as well as asking how the concept of teleonomy impinges on research at the frontiers of evolutionary science.

The analysis we offer is deflationary. We regard teleonomy as a somewhat curious artifact of mid-20th century “teleophobia,” as the product of a group of biologists working to distance themselves from the bad old days of Hans Driesch and Henri Bergson. During this period, many forms of teleological thinking flourished, including some we now regard as illicit. Yet while teleonomy achieves a certain separation between “good” and “bad” teleology, with teleonomy standing in for whatever forms of teleology pass scientific muster, it is not clear that the concept has much to offer ongoing inquiry. Rekindled enthusiasm for the concept therefore strikes us as misplaced. This is not to say that the activities of organisms have nothing to do with evolutionary outcomes; it is simply to question whether teleonomy provides useful resources for thinking about how this works, and for articulating its significance for evolutionary theory.

A quick note on terminology. The word “teleology” has Greek roots: *télos* (τέλος), meaning “goal” or “purpose,” and *lógos* (λόγος), meaning “an account or explanation.” “Teleology” therefore refers to explanation by goals or purposes, although the term is also used to refer to phenomena that seem susceptible to teleological explanation.^2^ “Teleonomy,” by contrast, combines *télos* with *nomos* (νόµος), meaning “law or custom.” It therefore suggests a union of lawfulness and purposiveness, or perhaps the scientific study of this union. Few themes in the history of biological thought are more prominent than the tension between lawfulness and purposiveness, since an explanatory style that appeals to laws or mechanisms seems to differ categorically from one that appeals to purposes. But this tension is in some senses illusory, and this has significant implications for what we make of teleonomy as a concept.

## A Little History of Teleological Thinking

### Greek Prelude

Teleological reasoning is probably as old as any activity recognizable as biology. When faced with a complicated and apparently useful structure, it makes sense to ask, “What is this structure (good) for?” To give a positive answer to this question is to engage in teleological reasoning. However, teleological reasoning has never been a single thing, and already in the 4th century bce a fault line had developed that marked a major split in the history of thinking about purposes (Lennox and Kampourakis 2013). This was the split between Plato and Aristotle, and like many splits between these two figures, this one would reverberate across subsequent millennia.

In his dialogue, the *Timaeus*, Plato defends the idea that the natural world is the product of an intelligent craftsman working to bring about the best possible ends (Lennox 1985; Johansen 2020).^3^ The conception is a teleological one. According to this view, the “Demiurge” (an artisan-like creator), “wishing all things to be good and nothing to be bad in so far as possible, took over everything which was visible…and led it from disorder to order, judging this to be in all respects better” (*Timaeus* 68e4–7). Central to this picture is the idea that the Demiurge imposes order on the world and its constituents from without, in accordance with “his” own conception of the good (Scolnivoc 2017). He accomplishes this by persuading materials to operate together for particular ends—although Plato allows that some changes are chaotic and differ categorically from those wrought by intelligence. As many have noted, these ideas resemble those operative in later natural theology, exemplified in such classic works as John Ray’s *The Wisdom of God Manifest in the Works of Creation* (1691) and William Paley’s *Natural Theology* (1802). However, it would be wrong to trace the entire tradition culminating in modern natural theology to a source in the *Timaeus* (McGrath 2011; Sedley 2017). Natural theology is more than Christianized Platonism, just as the account offered in the *Timeaeus* is more than an anticipation of later Christian theology.

Aristotle also defended the view that the world must be understood teleologically, at least in part. But this is just about the only similarity between his view and Plato’s. To begin, Aristotle deployed his teleology most energetically in the domain of living things (Lennox 2001). Probably its main use was to explain why animals have the parts and undergo the changes they do. To formulate such explanations, Aristotle enlisted the assumption that “nature does nothing in vain, but among the possibilities always does what is best for the being of each kind of animal, so that, if it is better in a certain way [for an animal’s being], that is also how it is according to nature” (704b14–17).^4^ This is not because he supposed nature to be the product of intelligent craftsmanship: he did not. Instead, his reasoning is based on the idea that each animal has needs that determine what is good for it in relation to its way of life.^5^ It is because an animal has these needs that it possesses the parts and undergoes the changes that it does. If it had different needs, it would have different parts and undergo different changes (that is, it would be a different kind of thing). At the base of all of this is the concept of “form,” which Aristotle took to explain why animals are the kinds of things they are. So, nature provides what is best for each kind of animal in relation to its needs, constrained by the properties of available materials.

In the secondary literature on teleological reasoning, it is customary to refer to Plato’s version of teleology as “extrinsic” or “external” and to Aristotle’s as “intrinsic” or “immanent” (see, e.g., Lennox 1992). These terms are useful because both flavors of teleology have been continuously present since antiquity. However, the interpretation and significance of teleological thinking has been subject to perennial dispute and negotiation, especially following the resurgence of mechanistic philosophies in the 17th century. Two developments have focused these disputes: the spread of mechanistic models of living things and the introduction of Darwinian evolutionary theory. In the remainder of this section, we provide a highly selective and condensed digest of these hugely complicated developments.

### Teleology in a Mechanical Universe

Although mechanistic approaches to living systems date to ancient Greece (Berryman 2009), it was not until the early modern period that these approaches began to garner serious attention from natural philosophers (Dijksterhuis 1961; Ruse 2003). One of their most influential proponents was René Descartes, whose description of nonhuman animals as *bête machines* epitomized an entire research program: to explain the development and workings of the body in terms of matter in motion and nothing else (Des Chene 2001). Aristotelians of the time preferred to explain these features in agential terms, as functions of a *psuchê* or “soul” that inheres in bodies of particular kinds. Yet Cartesian biology was predicated on a rejection of Aristotelian metaphysics and the entire edifice of reasoning it held up (Garber 1992; Newman 2001). This included Aristotle’s “intrinsic” teleology, which nonetheless continued to find employment in the study of medicine and comparative anatomy throughout the early modern period, most conspicuously in the work of William Harvey and his teachers in Padua (Lennox 2017b).

It is always dangerous to generalize, but it might be observed that a tension existed in early modern natural philosophy between the metaphysical demands of intrinsic teleology and a commitment to a mechanical universe. Nowhere is this more pronounced than in the work of Robert Boyle, a self-described mechanical philosopher who was committed to the destruction of Aristotelianism in all its guises. It was in no small part Boyle’s antipathy to Aristotle that spawned the myth that the Scientific Revolution saw a rout of teleological reasoning across the natural sciences. (Descartes helped advance this myth as well.) Still, Boyle evidently saw no tension between belief in a fully mechanical universe and the demands of a Christianized *extrinsic* (or Platonic) teleology. As he writes in his celebrated *Disquisition About the Final Causes of Natural Things*:

The most wise and powerful Author of Nature, whose piercing sight is able to penetrate the whole universe, and survey all the parts of it at once, did at the beginning of things, frame things corporeal into such a [mechanical] system, and settled among them such laws of motion, as he judged suitable to the ends he proposed to himself in making the world. (Boyle 1688, p. 91)

Among these “ends…in making the world” is the production of organic beings whose intricacy testifies to the agency of a creative intelligence: “For there are some things in nature so curiously contrived, and so exquisitely fitted for certain operations and uses, that it seems little less than blindness…not to conclude, that…they were designed for this use” (Boyle 1688, pp. 15–16).^6^ Similar arguments were made for the next two centuries under the aegis of natural theology, culminating in Paley’s watchmaker argument (McGrath 2011).

It is impossible to broach the subject of “mechanism” in biology without also mentioning its antagonist: “vitalism.” This is because the two movements were intertwined on a definitional level, with mechanists defining themselves in contrast to vitalists and vitalists returning the favor (Allen 2005). Roughly, vitalism is the idea that living things cannot be wholly understood in physicochemical terms, such that any purely mechanical analysis of a biological thing is bound to leave something out (Lovejoy 1911). For example, the embryologist Caspar Friedrich Wolff (1734–1794) thought it was impossible to explain embryogenesis without recourse to an organizing force (*vis essentialis*), which is “the initiating power of all processes of generation in organic beings” (Goy 2014, p. 45). Similarly, Hans Driesch, working around the turn of the 20th century, appealed to the notion of *entelechy* to explain the apparently goal-directed nature of developmental processes (Driesch 1908).^7^ Figures like Wolff and Driesch illustrate that intrinsic teleology was never rooted out of biological practice, despite the success of more mechanistic forms of analysis. Embryology, in particular, provided a refuge for more Aristotelian forms of teleology well into the 20th century, associated not only with vitalism but also with various forms of “organicism,” which shared with vitalism an interest in teleological phenomena while abjuring vitalism’s metaphysical adventures (Allen 2005; Esposito 2014; Peterson 2016).

Much more could be said about the long conflict between mechanism and vitalism, but we will limit ourselves to two remarks. First, vitalism had a long reach in the history of biology, partly because researchers found it difficult to imagine how apparently purposive processes like development could be adequately explained in physicochemical terms (Normandin and Wolfe 2013; Zammito 2018). This incredulity extended well into the 20th century, even though the number of card-carrying vitalists within professional biology had shrunk to almost zero by the time “teleonomy” appeared on the scene. Second, vitalists were disposed to regard biological phenomena as intrinsically purposive or as connected with purposive agencies like the *vis essentialis* or *entelechy*. This inclined them to adopt a broadly Aristotelian perspective that suffered considerably when vitalism dropped out of fashion and eventually plunged into disrepute.^8^ Because of this, intrinsic teleology remained much maligned, and much misunderstood, into the middle decades of the 20th century—a point that is useful for understanding both Pittendrigh’s motivation for introducing “teleonomy” and its immediate reception.

### The Darwinian Legacy

The second development that shaped the interpretation of teleology in biology was the introduction of Darwinian evolutionary theory. Prior to this, (extrinsic) teleology had been a central feature of the intellectual tradition known as natural theology, exemplified by Paley’s watchmaker argument. This argument holds that the existence of apparently well-designed entities in nature implies the existence of a designer capable of bringing such entities into existence (De Cruz and De Smedt 2014; Sedley 2017). Paley even suggests that an intelligent Creator is *necessary* to explain the appearance of purpose in the natural world because no other cause is sufficient to produce the effect. However, as Darwin showed in *On the Origin of Species* (1859), another explanation exists. If organisms vary in heritable characteristics, and if some of these variations make a difference in the struggle for life, then a process of “natural selection” can bring about the appearance of design in the organic world without the intervention of a deity. This is often held to have gutted the argument from design, and with it to have rendered obsolete all talk of purposes in nature.^9^ However, the advent of Darwinian theory in fact left teleological reasoning in an exceedingly complicated and ambiguous place.

For one thing, Darwin’s own position on teleology is controversial (e.g., Lennox 1993, 1994; Ghiselin 1994). In a much-quoted passage from 1874, the American botanist Asa Gray wrote that “Darwin’s great service to natural science” consisted in “bringing it back to Teleology; so that, instead of Morphology vs. Teleology, we have Morphology wedded to Teleology” (Gray 1874, p. 81).^10^ Darwin replied in a letter that this remark “pleases me especially”—evidently no one else had noticed his service to “Teleology.” But this is a curious remark from the man whose theory is said to have dealt teleology a killing blow. As Ghiselin writes (trying to resolve the apparent contradiction), Darwin’s “use of teleological metaphors in a strictly teleonomic context is irrelevant to the meaning of his discourse” (Ghiselin 1994, p. 489).^11^ In other words, Darwin was not a teleologist, notwithstanding that he sometimes helped himself to teleological language for reasons of convenience. Yet this response is unsatisfactory. What Gray understood was that Darwinian theory legitimized a certain appeal to “final causes” in nature; in Gray’s words, “usefulness and purpose come to the front again as *working principles* [in natural history]” (Gray 1876, p. 294; emphasis added). But if someone is utilizing notions of usefulness and purpose in their research, they are reasoning teleologically: and Darwin did, and was. Hence, in an important sense, Darwin *was* a teleologist (Lennox 1993).

There is more. While it may seem clear in hindsight that Darwin rehabilitated a form of teleological explanation (e.g., Ayala 1970; Brandon 1981), this was not so obvious to his contemporaries. In fact, in the decades following Darwin’s death, the acceptability of teleological explanation came under increased scrutiny, as first evolutionary morphology and then experimental biology drifted into vogue (Bowler 1996; Magnus 2000). During this time, and despite Darwin’s insistence that organic evolution contains no inherent goal or drive, theories of directed evolution gained influence while confidence in natural selection ebbed away (Bowler 1983, 2001). These theories were deeply teleological, but the teleology involved was not the sanitized extrinsic teleology of Darwin, nor was it the naturalistic and functional teleology associated with Aristotle. Instead, it was the rather different idea that *evolution itself* is a teleological process, self-directed by life towards greater complexity and, perhaps, a worldly apotheosis in man. Biologists and philosophers flocked to this “evolutionary teleology,” none more influentially than Henri Bergson, who married finalism about evolution to vitalism in the guise of the *élan vital* (Bergson 1907). And then there was the evolutionary theory of Pierre Teilhard de Chardin, composed in the 1930s (but only published after his death in 1955). On the eve of the modern evolutionary synthesis, then, teleology was far from extinct in any of its historical forms. Indeed, in an important respect, it was thriving.

## Enter “Teleonomy”

### Pittendrigh Introduces “Teleonomy”

To understand the introduction and uptake of “teleonomy,” it is useful to keep in mind the multiple meanings of “teleology” and their interactions with key developments in biological thought during the preceding decades. These developments include the complex position that Darwin left teleological reasoning in, not only because of his own ambiguous stance towards teleology, but also because of the success of new forms of evolutionary teleology in the years following 1859. After the *Origin*, traditional forms of teleology involving a craftsman-like Creator quickly lost scientific credibility. However, newer forms of extrinsic teleology that focused on the cosmos as a whole and invoked natural selection as a “secondary cause” became attractive options for many thinkers (Bowler 2001). At the same time, intrinsic teleology retained a foothold, as evidenced by the popularity of vitalism and organicism around the turn of the century (Esposito 2014; Peterson 2016). The latter positions were regarded with suspicion by much of the scientific mainstream because of their “extensive use of teleological explanations,” as well as their occasional association with evolutionary teleology (Esposito 2011, p. 18). But they remained a visible part of the landscape. Partly for this reason, the study of adaptation was deemphasized in large sections of biology, reaching “perhaps its lowest ebb of respectability about fifty years or more after *The Origin of Species*” (Pittendrigh 1958, p. 393)^12^

By the late 1950s, much had changed. The argument from design had mostly vanished from scientific discourse, even in its sophisticated post-Darwinian varieties.^13^ Vitalism too was nearly extinct and had seemingly taken Aristotelian teleology down with it. Evolutionary teleology had fared somewhat better, surviving both in the form of a general belief in progress and in nonstandard evolutionary theories like that of Teilhard (Ruse 1996). However, such theories were no longer the greatest threat facing newly professionalized evolutionary biologists, who found themselves in competition with molecular biologists for institutional positions, media attention, and funding opportunities (Beatty 1994; Milam 2010a). Partly in response to this situation, interest in adaptation had surged as researchers sought Darwinian explanations for a wide range of biological phenomena (Gould 1983; Milam 2010b). This provided the occasion for two symposia organized by Anne Rowe and George Gaylord Simpson, which culminated in the 1958 publication of *Behavior and Evolution*, containing Pittendrigh’s “Adaptation, Natural Selection, and Behavior.”^14^

Although Pittendrigh’s chapter is remembered for its coinage of “teleonomy,” his discussion is not an extended meditation on teleology in biology. Instead, it is a wide-ranging exploration of evolutionary adaptation, a subject that Pittendrigh observes, “has not received the explicit attention it merits in the large modern literature on evolution” (Pittendrigh 1958, p. 390). “Leaders in the modern phase of evolutionary thought,” most of them drawn from the disciplines of genetics and systematics, “seem in retrospect to have been preoccupied with the dynamics of population diversification at a highly abstracted level.” However, while “[it] is true that speciation involves (surely always [!]) the development of new adaptation, [to] leave it at that is not enough” (1958, p. 391). There are “quite fundamental aspects of the adaptation problem that transcend the short-range processes of population diversification and reflect a long historical chain of transient opportunities and conjunctures of opportunities as the truly creative agent responsible for contemporary biological organization.” It is this observation that supplies the central theme of Pittendrigh’s chapter and launches him on a discussion of “Teleonomy versus Teleology” in evolution (1958, pp. 391–394).

The discussion commences with a rundown of several meanings of “adaptation” in the biological literature, like the fit between organism and environment or “some feature of the organism…which serves a proximate end (food getting, escape, etc.)” (Pittendrigh 1958, p. 392). Collectively these indicate the “essential” feature of adaptation: “that aura of design, purpose or end-directedness which has, since Aristotle, seemed to characterize the living thing, [and] to set it sharply aside from the nonliving.” According to Pittendrigh, this aura has been adaptation’s

greatest burden in the history of biology. For adaptation as a genuine scientific problem was obscured up to 1859 by its association with Aristotelian teleology; and since 1859 it has had a hard time shedding a guilt acquired by that former association. (1958, p. 393)

By “Aristotelian teleology,” Pittendrigh seems to mean the idea that purposes are directly implicated in producing their effects—that fish have swim bladders because they need them to maintain neutral buoyancy. But this is muddled, Pittendrigh thinks, because final causes are not “materially efficient”: they are not part of the causal story concerning the origin of traits.^15^ A close reading of Aristotle reveals that he made no such claim. To have done so would have been to confuse the categories of final and efficient causation (to mix up reasons for existence and productive causes)—and these Aristotle was careful to keep apart. Still, for all Pittendrigh’s confusion, he does identify a blind spot in Aristotle’s biological program. To say that fish have swim bladders because they need them to achieve neutral buoyancy is to ignore the question of why fish need to regulate their buoyancy in the first place.^16^ And is this requirement really independent of the existence of organs like the swim bladder? Arguably it is not. So, Aristotelian biology seems to presuppose some of what contemporary biologists wish to explain. (To put the matter crudely, for Aristotle forms are given, for modern biologists they are derived.)

Turning his attention to Darwin, Pittendrigh claims that, “the concept of adaptation was by no means finally clarified, nor rescued from the disrepute of [‘Aristotelian’] teleology [in 1859]” (Pittendrigh 1958, p. 393). This is because “[the] concepts of adaptation and natural selection are so interwoven that it is impossible to misunderstand one without doing violence to the other.” But, Pittendrigh observes, neither concept was well understood in the 19th and early 20th centuries. Thus, “in the absence of an acceptable (nonteleological) explanation for [the] origin [of adaptation],” many students seem to have “solved the problem of adaptation by nearly denying its existence” (1958, p. 393). Yet with a better understanding of natural selection and a more precise concept of adaptation, the situation has since greatly improved. The question thus arises whether teleological language “should be resurrected” to refer to apparently end-directed phenomena in biology. Pittendrigh’s answer is a resounding no. “The biologist’s long-standing confusion would be more fully removed if all end-directed systems were described by some other term, like ‘teleonomic,’ in order to emphasize that the recognition of goal-directedness does not carry a commitment to Aristotelian teleology as an efficient [sic] causal principle” (1958, p. 394). Teleonomy thus entered the biological lexicon as a synonym for goal-directedness that avoids making the supposedly Aristotelian mistake that goals cause things to happen directly.

The remainder of Pittendrigh’s essay is a fascinating mishmash of themes and examples, from a nascent articulation of the gene’s eye view of evolution (“in a very real sense the developed organism is no more than a vehicle for its genotype” (1958, p. 398)) to a reflection on evolutionary contingency (“adaptive organization…[is] a patchwork of makeshifts pieced together…from what was available when opportunity knocked, and accepted in the hindsight, not the foresight, of natural selection” (1958, p. 400)). These are individually interesting, especially his suggestion that the gene’s eye view can be used to identify “evolutionary goals” for organisms and their parts. However, the most relevant feature for present purposes is Pittendrigh’s suggestion that organization per se (including biological organization) is intrinsically goal-directed. This has the effect of securing for teleonomy a maximal scope in the study of organisms and their activities. In line with post-WWII adaptationism, and consonant with themes in British natural theology, Pittendrigh assumes that to be alive is to be adapted, which in turn suggests that organismal biology can be understood as the study of adaptation in the broadest sense. But that is not all. The idea that teleonomy is a feature of biological organization also yields a restriction. By this criterion, evolution *itself* does not count as a teleonomic process. Evolution is a matter of populations, not individuals, and populations do not exhibit goal-directed organization. It follows, as a later popularizer of teleonomy would say, that “[it] is illegitimate to describe evolutionary processes or trends as [teleonomic]” (Mayr [1974]^1988^, p. 60).

### Teleonomy Spreads

Ernst Mayr was perhaps the first biologist to take up Pittendrigh’s concept of teleonomy and situate it in a different context.^17^ He did this in his seminal essay, “Cause and Effect in Biology” (Mayr 1961), which is usually remembered for introducing the distinction between “proximate” and “ultimate causation,” but which also contains a lengthy discussion of teleology in biology.^18^ The discussion begins by disavowing “[the] many dualistic, vitalistic, and finalistic philosophies of the past,” which made a mystery of goal-directedness and a hash of its explanation (Mayr 1961, p. 1503). But, Mayr observes, it remains wholly “legitimate to speak of purpose and purposiveness in nature” when the entity in question “has been *‘programmed’* [to] act purposefully” (emphasis added). Historical processes like evolution can never act purposefully because there is no informational program to guide the process towards a pre-set goal. Yet “[a] bird that starts its migration, an insect that selects its host plant, an animal that avoids a predator…all act purposefully because they have been programmed to do so” (1961, pp. 1503–1504). There is no intentional agency involved in this purposiveness; “it is a purely mechanistic purposiveness” governed by natural selection (1961, p. 1504). But it is purposiveness nonetheless, mediated by a well-adapted genetic program.

It is here that Mayr enlists the term “teleonomic.” Like Pittendrigh, Mayr’s goal is to draw a line between legitimate and illegitimate forms of teleology in biology. (For Mayr, “teleology” seems to mean little more than “goal-directedness.”) But unlike Pittendrigh, the key consideration for Mayr is whether an ostensibly goal-directed process is governed by a program or code of information. If it is, then that process can be regarded as a legitimate target of scientific inquiry. By contrast, vitalistic or finalistic processes, ideas about “the over-all harmony of the natural world” (echoes of Plato and the natural theologians), and proposals that evolution itself is goal-directed (“evolutionary teleology”) all come out as illegitimate (Mayr 1961, p. 1504). Mayr opts for “teleonomy” to designate the legitimate kind of goal-directedness because “teleology” has too many untoward connotations. However, unlike Pittendrigh, Mayr is inclined to restrict the designation “teleonomic” to processes and behaviors as opposed to structures or biological organization as such. This restriction is made explicit in his later writings (e.g., Mayr 1974).

Mayr wrote two major articles on teleonomy in 1961 and 1974. In between, two celebrated works appeared that featured the word “teleonomy” in prominent positions. The first was *Adaptation and Natural Selection* by George C. Williams, in which “teleonomy” was used to name a branch of study concerned with the question, “What is [a trait’s] function?” (Williams 1966, p. 258).^19^ Needless to say, this application of the term has not caught on, even though it also rears its head in Richard Dawkins’s influential book, *The Extended Phenotype* (1982). The second celebrated work to feature “teleonomy” was Jacques Monod’s *Chance and Necessity*, published in 1971. Here “teleonomy” refers not to a field of study but to “one of the fundamental characteristics common to all living beings without exception: that of being *objects endowed with a purpose or project*” (Monod 1971, p. 9; emphasis in original). Monod defines what he calls the “essential teleonomic project” as “the transmission from generation to generation of the invariance content [or teleonomic information] characteristic of the species” (1971, p. 14). But this is somewhat misleading if it is taken to mean that teleonomy is mostly about inheritance. Instead, Monod observes that the transmission of teleonomic information “calls assorted, more or less elaborate and complex structures and performances into play” (1971, p. 15), and these too can be described as teleonomic, even relatively static structures, like bits of anatomy. It was this suggestion that prompted Mayr to complain that Monod’s “teleonomy” is indistinguishable from the concept of evolutionary adaptation as such (Mayr 1974)—a curious complaint given that the same might be said of Pittendrigh’s original treatment.

In any event, Mayr’s observation that “teleonomy” had become overextended suggests a reason for its marginalization following its 1970s heyday. For Monod, teleonomy names the fundamental “project” of living systems: their mechanical pursuit of self-reproduction, supported by a range of functional adaptations. For Pittendrigh it means something similar, and also for Mayr, except with the condition that this pursuit is associated with the running of a genetic program. Williams used “teleonomy” to name a serious science of adaptation, which he took to be lacking in the mid-1960s, and Dawkins (1982) followed him. All these usages made sense when the concept of adaptation had a certain unscientific glow, as it did in the early-to-mid 20th century. However, as battles over vitalism and the meaning of evolutionary adaptation receded, the need for a concept that affirms the scientific status of adaptation became less pressing. The term consequently receded from prominence as new generations of biologists turned their attention to new opponents and new battles. These included a battle over just how prevalent adaptation is: a battle some versions of “teleonomy” made trivial by defining biological organization itself as adaptive (Gould and Lewontin 1979; Mayr 1983; Cain 1989; Pigliucci 2000).

But that is not all that happened during the latter decades of the 20th century. In addition, philosophers—including philosophers working in the new specialty of philosophy of biology—mounted an influential campaign to rehabilitate teleological explanation within a broadly naturalistic framework. This cast doubt on the need for a term that describes the property of goal-directedness while avoiding all association with “the debased currency of teleology” (Simpson 1958, p. 521).

## Back to the Future

### The Rehabilitation of Teleological Explanation

In our preceding discussion, we mostly ignored the period from about 1930 to 1950. This was the period of the evolutionary synthesis, but it also was the period that saw the first flowering of cybernetics, which is highly significant for discussions of teleology. Cybernetics set out to analyze purposive systems, especially (but not limited to) human-made ones like self-regulating machines or “servo-mechanisms.” It therefore cried out for an analysis of teleology, and as the field found its footing in the 1940s, teleology was indeed a subject of discussion (e.g., Rosenblueth et al. 1943). A common strategy in these discussions was to begin with a behavioral definition of teleology, such as behavior exhibiting “persistence” and “plasticity.” Explanatory accounts would then try to show how the processing of information by systems with a particular organizational structure enables them to exhibit the relevant behavioral features (Simon 1976).

This approach to teleology exerted a wide and pervasive influence during the middle decades of the 20th century. However, it did not provide the basis for a more general rehabilitation of teleological *explanation*. This only commenced in the 1970s when philosophers interested in the logic of function statements and the nature of biological adaptation began formulating new accounts of teleology and teleological explanation (e.g., Ayala 1970; Ruse 1971; Wimsatt 1972; Wright 1973, 1976). An important conclusion that emerged from these analyses is that standard selectionist explanations for the existence of traits are a kind of teleological explanation (Brandon 1981). This is because they explain the existence of traits by pointing to something those traits do (or at least something that past tokens of those traits did). Put schematically: some *X* exists because it does *Z* (Wright 1976).^20^ This is a characteristically teleological form of explanation, and yet it involves no violation of scientific norms, like the injunction that causes must precede their effects in time. The reason is that the effects that explain their causes in a selectionist explanation are the past effects of earlier tokens of those causes. Selectionist explanations are therefore causal, historical, and teleological at the same time.

To see the importance of these remarks for the status of teleonomy, it is useful to highlight an ambiguity in contemporary uses of “teleology.” On the one hand, teleology refers to a *form of explanation* characterized by what has been termed “consequence etiology”: some item exists because it brings about a consequence identified as its goal, purpose, or function (Wright 1976). On the other, teleology indicates a *phenomenon to be explained*, usually, but not always, meaning goal-directedness. (This was how the cybernetics movement understood “teleology.”) The term “teleonomy” entered the biological lexicon as a synonym for teleology in the second sense. For Pittendrigh (1958, p. 394), it permitted “the recognition and description of end-directedness [in living systems],” while steering clear of any commitment to the validity of teleological explanation. This likely recommended the term during a period when teleological explanation was widely misunderstood; witness Mayr’s (1961, p. 1502) strange argument that when evolutionary biologists ask “*why* [does some feature exist],” they do not intend to ask “the finalistic *‘what* [is this feature] *for*’” question, but instead “the historical ‘*how come* [this feature exists]’” question. But if the way one answers a “finalistic ‘*what for*’” question is by answering a non-finalistic “*how come*” question (as modern analyses of selectionist explanations suggest), then there is no reason to avoid the finalistic question. This removes some of the motivation for having a term that means “goal-directedness,” while at the same time conveying an uneasiness about teleological explanation: at least when teleological explanation is understood along the lines indicated above.

What does this mean for “teleonomy”? Assuming philosophers are correct that certain teleological explanations are scientifically acceptable, biologists no longer require terminology that signals a blanket agnosticism towards teleological explanation. This was once an important element of teleonomy’s appeal; in Thompson’s words, Pittendrigh coined the term teleonomy “to free [the study of goal-directed processes] from the encumbrances of teleological explanation” (Thompson 1987, p. 259). But the situation today is different, and to the extent that “teleonomy” continues to perpetuate the idea that teleological explanation is disreputable or unscientific, it arguably does a disservice to clear thinking about purposes in biology.

Still, this does not mean that “teleonomy” has no value. There is a persistent ambiguity in the biological and philosophical literature between the two senses of teleology: teleology as a form of explanation versus teleology as a phenomenon to be explained. To the extent that “teleonomy” succeeds in picking out the latter, it may have a role to play in clarifying discussions of goal-directedness in biological systems. This would be the case especially if a notion of teleonomy could be agreed upon that succeeds in drawing a line around those processes that can legitimately be described as goal-directed and those that cannot. It no longer seems that Mayr’s appeal to genetic programs will do the trick (Moczek 2012), but perhaps there is a way of explicating teleonomy that moves beyond US Supreme Court Justice Stewart’s exasperated reply, “I know it when I see it.”^21^

Yet even here it is not clear that an intervention is necessary. In our estimation, the ambiguity between the two senses of teleology, while real and often unnoticed, causes little mischief in scientific discourse. It is almost always clear from contextual clues whether an author is referring to teleological explanation or goal-directedness as a phenomenon. This suggests that biologists do not *need* a word like “teleonomy” to extricate themselves from a semantic predicament; to speak of “teleology” (or if one is squeamish, “apparent teleology”) will cause few problems for careful language users.

In summary, we regard teleonomy as an amorphous and arguably unnecessary concept. Of these concerns, it is the former that is the more serious. While all proponents of teleonomy agree that it attaches to the activities (and possibly the parts and general organization) of biological systems, its exact extension is unclear. What kinds of systems are organized to exhibit teleonomic activities? How do we decide which of the activities performed by organisms are properly teleonomic? And if answers to these questions involve reference to “goals” or “goal-directedness,” how are we to understand these concepts? Currently there is no consensus on what “goal-directedness” means in biology, and the notion of a “[biological] goal” is philosophically elusive and controversial (but see Lee and McShea 2020 for a recent suggestion). “Teleonomy” inherits all these difficulties, which makes it hard to see what it stands to contribute to their clarification.

### Whither Teleonomy?

So far, this analysis has been mostly negative. “Teleonomy” offers little that is urgent and distinctive for biology. In fact, we have argued that it is basically a synonym for goal-directedness with a contested extension. It gained popularity in the 1960s and 1970s as organismal biologists grappled with the complex legacy of teleological reasoning in biology. This involved at once distancing themselves from teleological explanation, while at the same time affirming the existence of a domain of goal-directed processes associated with organized systems or genetic programs. Yet after a short heyday it foundered, partly because it became overextended, and partly because new philosophical work dispelled some old stigmas about teleological explanation.

But this is all history. What about the future? Recent years have seen increased interest in the possibility of a revised and expanded evolutionary theory (e.g., Laland et al. 2015; Müller 2017; Jablonka and Lamb 2020). A significant amount of this interest surrounds the theme of organismal agency in evolution (Walsh 2015; Diogo 2017; Sultan et al. 2022). Since “teleonomy” refers to the goal-directed activities of living things (or else to their basic quality of purposiveness), it seems that this concept could have some role to play in these discussions. Consider that teleonomy is clearly related to agency. Thus, to interrogate the active role of organisms in evolution could be seen as exploring the evolutionary significance of teleonomy (Corning 2014).^22^ But here again we confront the problem that the explanatory gains provided by teleonomy fail to counterbalance its unclear extension and general fuzziness. What benefits accrue from talk of “teleonomy” that are unavailable with talk of behavior, phenotypic plasticity, niche construction, and extended inheritance? Or, to put the question somewhat differently: what reason do we have for thinking that there is anything general and interesting to say about the role of teleonomy in evolution?

One possible reason has to do with Mayr’s proximate/ultimate distinction. As Corning (2019, p. 913) observes, Mayr draws a sharp distinction between proximate and ultimate causation, which seems to “exclude proximate forms of causation from exerting a direct influence on ultimate causes (natural selection and evolution).” Since teleonomy belongs to the realm of proximate causation, it seems to follow that teleonomy can have no impact on the trajectory of evolutionary change. Teleonomy is a *product* of selection, not its *cause*. Corning seeks to undermine this conclusion by noting that proximate causes (including teleonomic ones), “are deeply and inextricably involved in all evolutionary change…proximate causes of various types, in interaction with a given environmental context, …are the underlying causes of natural selection” (Corning 2019, p. 914). The traditional formula is accordingly reversed: teleonomy is the *cause* of natural selection, not its passive *product*.

But would Mayr, or any traditional Darwinian, really deny this? It is hard to imagine a Darwinian biologist arguing that what organisms do is irrelevant to differential survival and reproduction; the struggle for existence may be metaphorical, but it is not *that* metaphorical. Likewise, no Darwinian would deny that functional advantages “drive” selection. It is not the purpose of the proximate/ultimate distinction to draw a line between functional effects as proximate causes and natural selection as the ultimate cause. Mayr wanted to show that there are multiple answers to questions like “why do swallows migrate south in the winter?” Some answers appeal to “proximate” factors like physiological cues; others appeal to evolutionary ones like the adaptive value of migration. Nothing in this formulation implies that goal-directed behaviors are irrelevant in evolution. In the migration example, it must be the case that selection is operating on variation in goal-directed behavioral programs (to use Mayr’s categories): what else could be going on? So, Corning is right that “proximate functional effects are [indeed] ultimate causes” (Corning 2019, p. 914), but for the uninteresting reason that goal-directed activities contribute to fitness.

A somewhat different concern is that mainstream evolutionary biologists have downplayed the importance of teleonomy in evolution, perhaps owing to a reliance on simplistic causal models. This concern has also been articulated in terms of proximate and ultimate causation. Laland et al. (2011) emphasize how proximate and ultimate factors can interact in complex ways to produce evolutionary outcomes; a good example is the ability of niche-constructing activities to generate evolutionary time lags. This is not the same as Corning’s claim that proximate causes (in interaction with environments) “are the underlying causes of natural selection,” which imputes to biologists a simple conceptual mistake. Instead, it is the claim that “proximate” factors sometimes modulate natural selection and vice versa, such that they partly explain evolutionary outcomes that simpler models only ascribe to selection. Notably, Laland et al.’s proximate causes include so-called “teleonomic” ones. So, these authors can be interpreted as arguing that the role of teleonomy in evolution has been underestimated, albeit for somewhat different reasons than Corning.

This strikes us as a legitimate concern. Although certain debates about the importance of teleonomic factors in evolution (e.g., niche construction) appear to involve conflicting sets of assumptions for evolutionary modeling (Scott-Phillips et al. 2014), there are genuine questions about what factors shape evolutionary trajectories at different spatial and temporal scales. Studies are emerging that suggest both continuity and disparity across a range of scales. For example, only recently have some studies indicated that standard evolutionary quantitative genetics is applicable to much larger timescales than were considered previously; standing genetic variation within a population of *Drosophila melanogaster* was strongly correlated with phenotypic divergence across forty million years of evolution in the Drosophilidae (Houle et al. 2017). However, once we move outside closely related lineages, evolutionary patterns appear to be conditioned more by historical depth, phylogenetic distance, and ecological differences than by patterns of genetic variation (Blount et al. 2018). Phylogenetically-informed macroevolutionary modeling arising from new models of trait and lineage evolution can facilitate the study of evolvability over long timescales, including questions about how lineage diversity and morphological disparity are regulated through time and what accounts for macroevolutionary stasis (Hunt and Slater 2016; Love et al. 2021). But much remains uncertain. In general terms, we lack an appropriate range of empirically discriminating models that speak for or against particular factors, teleonomic or otherwise, playing larger or smaller roles in shaping evolutionary trajectories at different scales.

This suggests that there is value in scrutinizing the evolutionary significance of those factors labeled “teleonomic” by Corning and others (e.g., Uller and Laland 2019). The relevant research questions engage a set of thorny issues about the relationship between microevolution and macroevolution that have been controversial for decades, and that remain underexplored (Erwin 2000; Leroi 2000; Abouheif 2008). However, our abbreviated historical review from ancient Greece to the 20th century, along with our survey of recent philosophical analyses of teleological explanation and goal-directedness, suggest that teleonomy, while mostly inoffensive, does little to facilitate clear thinking in terms of purposes. The shift from *lógos* to *nomos* offers no distinctive advantage when *télos* is under scrutiny, whether as a phenomenon to be explained or as a form of explanation. The deeply felt tension between lawfulness and purposiveness in living systems is less troublesome than it was made out to be, and any genuine legacy from discussions of teleonomy will likely appear in redoubled efforts to formulate new models that tease apart the relative significance of distinct factors in shaping evolutionary trajectories on different spatial and temporal scales.

## Footnotes

1 This conference was titled “Evolution ‘on Purpose’: Teleonomy in Living Systems,” and was organized by Peter Corning and Dick Vane-Wright. The title was drawn from an earlier article in the Linnean Society’s professional journal, written by Corning (2014). An edited volume based on the conference is forthcoming.

2 These are sometimes styled as “teleological phenomena,” other times simply as “teleology.” Importantly, not everyone who speaks of “teleology” as a phenomenon believes in the validity of teleological explanation. Some aim instead to show that (apparently) teleological phenomena can be explained without reference to goals and purposes (e.g., McShea 2012).

3 This is a common interpretation (Zeyl and Sattler 2022). In this dialogue, the main speaker is Timaeus (generally taken to be Plato’s mouthpiece), who describes his cosmology as a “likely account.” It is not entirely clear what this means, but Plato seems to regard the account as a plausible one.

4 This quotation is from Aristotle’s short work on animal locomotion, *De incessu animalium*.

5 Aristotle sometimes speaks of needs more positively, as “preferences and inclinations” (see Lennox 2017a). Being a carnivore is one such inclination, for which the associated need is the procurement and digestion of meat.

6 In contrast, “I never saw any inanimate production of nature, or, as they speak, of chance, whose contrivance was comparable to that of the meanest limb of the dispicablest animal” (Boyle 1688, p. 47).

7 “Entelechy” is an Aristotelian term meaning “having one’s purpose within.” It is associated with intrinsic teleology in contrast to the Christianized extrinsic teleology of Boyle and the physico-theologians.

8 Organicist biologists like E.S. Russell and W.E. Ritter kept the Aristotelian heritage alive during the first half of the 20th century, but remained outside the biological mainstream (Esposito 2014; Peterson 2016). Their marginalization owed partly to a suspicion that organicism was just warmed-over vitalism, a suspicion that proponents failed to outrun despite much outspoken criticism of vitalism’s excesses (Nicholson and Gawne 2015; Dresow 2020). Regardless, while organicists produced much thoughtful work on teleology and teleological explanation, this work failed to penetrate very far into the main currents of biological thought.

9 Illustrative is the remark of William James that despite “how unanimously our ancestors felt the force of [the] argument [from design]…Darwin opened our minds to the power of chance-happenings to bring forth ‘fit’ results if only they have time to add themselves together” (James 1907, pp. 110–111).

10 “Morphology,” for Gray, comprises “all inquiries into the structure, the parts, and the organic composition of vegetables,” as well as “the organs or obvious parts of which plants are made up” (Gray 1879, p. 2). “Teleology,” by contrast, is “the study of functions, or of the living being (animal or plant) in action…Every thing in the flower is in relation to fertilization and fructification, directly or indirectly” (1879, p. 216).

11 By “teleonomic,” Ghiselin has in mind something like Mayr’s meaning, in which apparent goal-directedness appears as a mechanical result of a program of information “programmed” into organisms by natural selection (see the “Teleonomy Spreads” section).

12 As Walter Garstang complained in 1898: “The vast majority of ‘biologists’ are almost exclusively engaged in the study of comparative anatomy and embryology. The amount of attention paid to these branches of biology has long been utterly out of proportion to the scant attention devoted to the scientific study of the habits or animals and of the function of the organs and parts composing their bodies” (Garstang 1898, pp. 211–212).

13 Religious scientists did not abandon the project of reconciling their scientific and religious beliefs with the downfall of creationist biology (e.g., Dobzhansky 1967). Still, by the middle of the 20th century, these efforts had been mostly excluded from the realm of professional discourse in evolutionary biology (Ruse 1996).

14 Colin Pittendrigh, often referred to as the “father” of circadian biology, was a student of Theodosius Dobzhansky and spent his career at Princeton and Stanford (Menaker 1996). His interests ranged widely, but were anchored in evolutionary considerations related to adaptation and biological rhythms.

15 The expression “materially efficient” is an unfortunate turn of phrase, especially as a description of final causes, because Aristotle distinguished four kinds of causes (or kinds of answers to why-questions), including the *final*, the *material*, and the *efficient*, as well as the formal.

16 At least this is a blind spot if one rejects explanations based on substantial forms.

17 Mayr’s first discussion of teleonomy appeared in 1961. Also in 1961, Cold Spring Harbor dedicated its annual symposium on quantitative biology to “Cellular Regulatory Mechanisms.” Both the opening and closing remarks (by Bernard Davis, and Jacques Monod and François Jacob, respectively) were framed in terms of teleonomy (Davis 1961; Monod and Jacob 1961). Still, neither contribution makes any attempt to unpack the meaning of teleonomy or to explore its extension, beyond gesturing at Pittendrigh. For this reason, we award priority in grappling with teleonomy to Mayr.

18 “Proximate causes” are the immediate or mechanical causes of traits, including behaviors presumed to be adaptive. “Ultimate causes” are captured by functional or adaptive explanations of the same traits (cf. Beatty 1994).

19 Williams thinks that progress in the study of adaptation demands standardized criteria for demonstrating adaptation along with “a formal terminology for its description” (Williams 1966, p. 260). He is skeptical of Pittendrigh’s claim that biologists can observe or otherwise intuit the proximate ends of specific structures: hence the need for an explicit science of adaptation (teleonomy).

20 And *Z* is responsible for the survival of *X*, or else the production of further tokens of *X*. So, the causal structure is a loop: *X*  > *Z * > *X * > *Z* ….

21 One possibility derives from a recent proposal that biological organization is “inherently teleological” (Mossio and Bich 2017). This proposal grounds teleology in the phenomenon of “self-determination” understood in terms of a closure of “constraints” (local and contingent causes that reduce the degrees of freedom of the dynamics on which they act). On this account, any activity that contributes to maintaining the closure of constraints in a biological system could be said to be “teleonomic,” meaning conducive to the maintenance of an intrinsically teleological/teleonomic regime of organization.

22 This way of framing things ignores the “active role” of constraints in channeling evolution down preferred pathways of change: a role of organisms in evolution stressed by Gould (2002). Here constraints are not understood teleonomically. Constraints are not oriented by any goal, but they can still play an active role in shaping the direction of evolutionary trajectories.
